# Tryptophan Levels during Grape Ripening: Effects of Cultural Practices

**DOI:** 10.3390/molecules22060941

**Published:** 2017-06-06

**Authors:** Ana Ruiz-Rodríguez, Ceferino A. Carrera, Widiastuti Setyaningsih, Gerardo F. Barbero, Marta Ferreiro-González, Miguel Palma, Carmelo G. Barroso

**Affiliations:** 1 Department of Analytical Chemistry, IVAGRO, Faculty of Sciences, University of Cadiz, Puerto Real 11510, Spain; ana.ruiz@uca.es (A.R.-R.); ceferino.carrera@uca.es (C.A.C.); widiastuti.setyaningsih@uca.es (W.S.); gerardo.fernandez@uca.es (G.F.B.); marta.ferreiro@uca.es (M.F.-G.); carmelo.garcia@uca.es (C.G.B.); 2 Department of Food and Agricultural Product Technology, Faculty of Agricultural Technology, Gadjah Mada University, Jalan Flora, Bulaksumur 55281, Yogyakarta, Indonesia

**Keywords:** tryptophan, grapes, ripening, cultural practices

## Abstract

Some cultural practices that are carried out during the grape ripening period are associated with vine stress, including leaf removal, grape bunch removal, and vegetable cover crops. Additionally, several nitrogen and sulfur supplements have also been used directly on leaves during the last stage of the ripening period. In the work described here, five different cultural practices and the reference were applied in three replicates in the same vineyard. The evolution of tryptophan levels was evaluated from just after grape veraison until the harvest date. In some cases, certain specific treatments were also evaluated after the regular harvest date. The cultural techniques that involved the application of nitrogen led to higher levels of tryptophan at the harvest day when compared to other cultural techniques. It was also found that the application of nitrogen without sulfur had a faster effect on the level of tryptophan. It was established that a period of around 20 days is needed for the grapes to show clear differences in tryptophan levels after the application of nitrogen.

## 1. Introduction

Several specific treatments applied to vine leaves had significant effects on the final composition of the resulting wine, including a marked enhancement of wine aroma. For example, foliar treatment with oak or extracts from vine shoots produced higher amino acid levels in must and different compositions of volatile compounds in wines [1]. The specific application of nitrogen to vine leaves in the vineyard has proven to be useful to increase the concentrations of the main nitrogen compounds in grapes, including total nitrogen, total amino acids, arginin, proline, and ammonium. This cultural practice results in higher levels of available nitrogen for yeast (YAN) during the alcoholic fermentation and has a direct effect on the quality of the final wine [2]. It has been reported that the addition of nitrogen increases the amino acid concentration in the Tempranillo variety along with urea and, in particular, phenylalanine [3], anthocyanins, and flavonols [4,5].

On the other hand, the amino acid levels in the grapes are strongly related to the maturation. The amino acid tryptophan is a precursor of indoleacetic acid (IAA), which is the active form of auxin phytohormone, and the content of this compound is conditioned by the grape maturation metabolism [6].

Ripening in the grape cluster is a complex process. The grapes enter maturation asynchronously and the process involves auxin and abscisic acid, with different kinetics observed depending on the berry weight and the number of seeds [7]. Furthermore, this process can be altered to delay harvest using reagents such as ethylene(2-chloroethyl)phosphoric acid [8] or 1-naphthaleneacetic acid [9].

A knowledge of the concentrations of auxin precursors, such as the amino acid tryptophan, in the maturation phase is of particular interest to provide information about the process and the effects of different cultural practices.

Various grape vines that are typical of cooler climates have started to be grown in areas with warmer climates. The ultimate goal of winemaking companies is the production of new wines in an effort to increase the regular markets. The use of new vine varieties in warmer areas requires new knowledge of the effects that cultural practices have on the final grapes. The Verdejo grape variety is well known in northern Spain for its quality wines. However, there is very little information on the cultivation of this variety in warmer regions. The area of Jerez (Spain) is very sensitive to rising temperatures and reduced rainfall. An understanding of the maturation of grapes in these weather conditions is of particular interest because it will be very similar to the conditions that may become common in the northern areas in years to come due to climate change [10,11].

In extreme temperatures, the metabolism in the vine is inhibited and this reduces the accumulation of metabolites, which in turn affects both the color and the aroma of the grape. Furthermore, after harvest and during winemaking, the high concentration of sugar in the grape juice leads to yeast stress, and this carries the risk of deviations that result in unwanted fermentation products such as acetic acid [12,13].

The application of appropriate cultivation techniques improves the phenolic ripening process of the grape and the aromatic potential [14]. Watering, pruning and thinning, amongst other techniques, play an important role in the performance of the vine and the balance between phenolic and technological maturity [15,16].

The technique of leaf removal close to the cluster allows suitable sun exposure to increase the glucose, grape polyphenol, and volatile composition of the wine. This technique has been used during the growth of Pinot Noir grapes and has had a positive effect on the final wine [17].

The amino acid contents of Verdejo grapes have been studied in relation to their maturity, and the results indicate that climatic conditions have an effect on the amino acid levels [18]. Similar conclusions were drawn in work with other varieties, with the differences depending on the use of a particular cropping system [19,20].

The study described here concerns the effects of five different cultural techniques and the reference on the levels of tryptophan from 100% veraison to the post-harvest season in the Verdejo grape variety cultivated in a warm climate.

## 2. Results and Discussion

**Figure 1:**
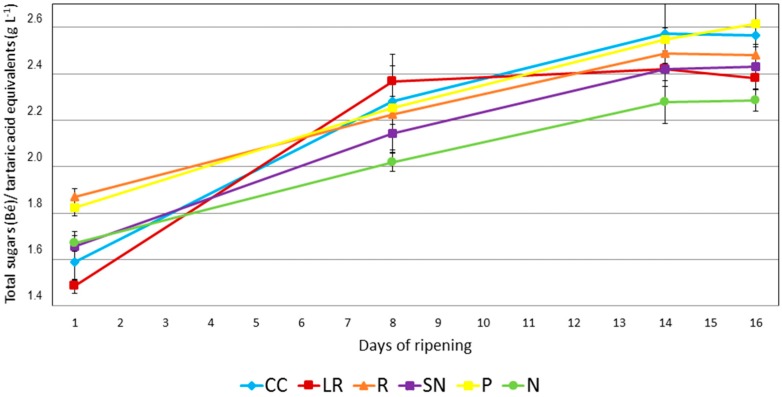
Evolution of the technological maturity (total sugars (Bé)/tartaric acid equivalents (g L^−1^)) during the ripening period for grapes from five different cultural practices and the reference (CC: Cover crops, LR: Leaf removal, R: Reference, SN: Sulfur and nitrogen addition to vine leaves, P: Specific pruning, N: Nitrogen addition to vine leaves).

One of the most widely used parameters to characterize the ripening period is the technological maturity, which is defined as the ration between the total sugars level (in Baumé units) and the total acidity (tartaric acid equivalent in grams per liter). Grapes from three different areas in the vineyard were studied during the 16 days before the harvest. It can be seen from Figure 1 that specific cultural techniques had a dramatic effect on the technological maturity. This behavior is consistent with the results of other research on the important effects that cultural techniques have on the yield of the vines and the quality of the resulting wines [14,15,16]. Measurements were made throughout the ripening period.

The two techniques used for the addition of nitrogen to leaves (N and SN) lead to lower maturity in comparison to the reference culture conditions at the end of the ripening period. It should be noted that, at the first point of the study, SN and N samples showed intermediate levels of ripening when compared to samples generated by the other cultural techniques. It can be supposed that 5–9 days after nitrogen/sulfur applications, the effects of nitrogen were not yet apparent on either the vines or the grapes. However, on day 8, i.e., 17 days after the first nitrogen application, N had a ripening degree of 2.0 and SN a value of 2.1, i.e., the lowest technological maturities of all the studied samples, which gave values in the range from 2.2 to 2.4. At day 14, N-type samples also had lower technological maturity, but SN-type samples showed similar values to those obtained by the reference (R) cultural practice and the same as the LR-type grapes. Similar results were also obtained on the harvest day. Differences between the sugar levels of the grapes from SN and N treatments and the R samples were also observed.

At the first point in the study, the grapes from the pruning treatment (P) group had a similar technological maturity to those from the reference cultural conditions. However, grapes from the cover crop treatment (CC) series had a lower technological maturity. After day 8, grapes from these three cultural conditions all showed very similar values. On the harvest day, P and CC assays gave higher values when compared to the other cultural techniques.

The leaf removal technique (LR) produced a much lower value at the beginning and later, after day 8, the value remained virtually unchanged at around 2.4. It has been reported that leaf removal close to the cluster provides better sun exposure, which in turn increases glucose, grape polyphenols, and the volatile composition of the wine [17]. In this study, some differences were observed at the beginning/middle of the ripening process, but at the harvest date the final technological level was quite similar to that of the R grapes.

**Figure 2:**
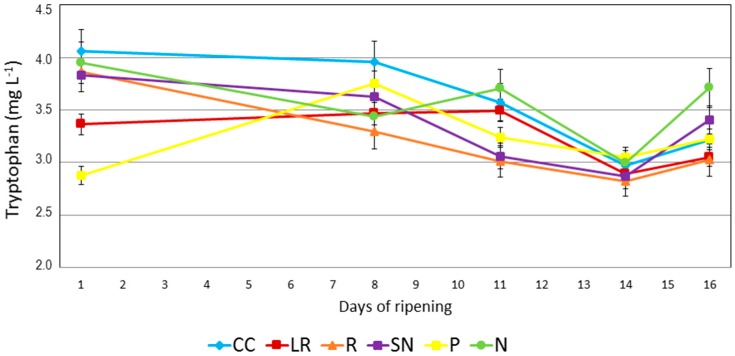
Evolution of tryptophan levels during the ripening period for grapes from five different cultural practices and the reference.

Regarding the evolution of tryptophan during ripening (Figure 2), it should be noted that the levels of tryptophan at the starting point of the study were clearly different between grapes from different cultural practices. It should also be noted that the differences were not related to the technological maturity levels of the grapes. It can be seen that the grapes with the highest technological maturity, i.e., R and P, at the first point of the ripening period had a technological maturity of 1.8 but did not have the lowest levels of tryptophan: R had a tryptophan content of 3.86 mg L^−1^ whereas P had a tryptophan level of 2.87 mg L^−1^. On the other hand, the cultural practices that gave rise to the lowest technological maturity (LR 1.49 and CC 1.59) did not contain the highest levels of tryptophan (LR 3.36 mg L^−1^ and CC 4.06 mg L^−1^). It should also be noted that N- and SN-type grapes had very similar tryptophan values to grapes from the reference cultural technique. As explained above, the nitrogen supplement was applied 9 and 5 days before first day of analysis, and it must therefore be concluded that an effect on the tryptophan levels is not produced in the grapes after that period.

In studies on other grape varieties, differences in the amino acids and nitrogen were observed depending on the cultural practice applied [19,20]. In any case, nitrogen supplements were always provided close to the veraison point because it is known that there is a delay before the plant can use the applied nitrogen and thus for the grapes to reflect the effects in terms of their composition.

It can be seen from Figure 2 that the cultural practices also have an effect on the tryptophan levels and the evolution of this compound during the ripening period. The tryptophan levels decreased for cultural practices N and R. By contrast, in the cover crop (CC) samples, the tryptophan level remained virtually constant during the main part of the ripening period, although a dramatic reduction was observed after day 8 until the final value was reached, which was around 27% lower than at the starting point. The leaf removal cultural technique (LR) gave rise to a constant maturation level with a value of 3.4 mg L^−1^. However, a clear reduction of 12% was determined at the harvest date and this value is similar to that obtained for the reference (R) (3.0 mg L^−1^).

Assays in which nitrogen was applied to the leaves (N and SN) showed different behavior to the other cultural techniques at the end of the ripening period. Firstly, during the first eight days, i.e., 17 days after the first nitrogen application and 13 days after the second application, these vines showed a non-specific different behavior when compared to the other assayed cultural practices, with either no change or a slight decrease in tryptophan levels observed during this period. However, a few days before harvest, the tryptophan levels started to increase in both cases. The N samples showed an increase of 7% in the period from day 8 to day 11, whereas the CC, P and R trials were lower by an average of 10% and LR remained constant. Subsequently, between days 11 and 16, the SN-type samples also showed an increase in tryptophan by 10%, while the results for the other trials remained constant or decreased. This effect was not observed for other cultural practices and it therefore must be related to the addition of nitrogen several days earlier in the process. In previously published papers it was reported that the use of nitrogen in the vineyard increased the concentrations of the main nitrogen compounds [2] and that different effects could be achieved by adding different nitrogen-based supplements to vines. For example, in the Tempranillo variety, the addition of phenylalanine has a greater effect than the addition of urea [19]. Therefore, the main effects caused by nitrogen supplements on grapes were recorded several days before the harvest point, i.e., around 20 days after the first application to vine leaves.

The harvest date is usually established on the basis of sugar and acidity levels, although it is also related to some changes in the evolution of the berry. After a specific ripening degree, berries start to lose weight due to water loss caused by high temperatures. This process becomes more important than the accumulation of compounds by the vine, i.e., grapes begin a conversion process to raisins, which have higher levels of sugars and lower water contents. This process could affect the evolution of tryptophan levels.

**Figure 3:**
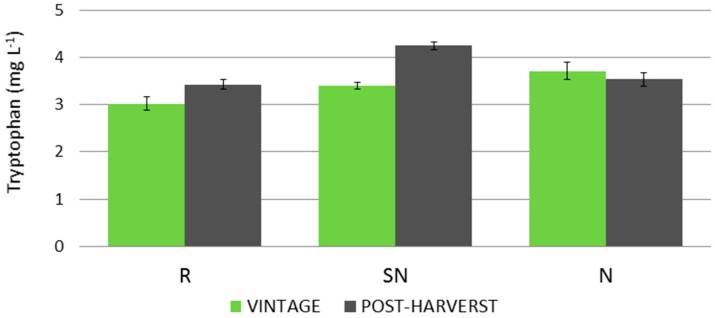
Evolution of tryptophan levels during the post-harvest period for three different cultural practices.

The markedly different evolution of tryptophan levels in grapes led to the selection of SN and N samples for a post-harvest study. For this purpose, an additional sample date was carried out for these cultural practices and for the reference culture (R) in order to compare the results with those for the untreated grapes/vines. The results are represented in Figure 3.

During the post-harvest period, grapes on vines produced higher levels of tryptophan for the SN and R cultural conditions, with increases of 19% and 12%, respectively, whilst N did not show any further evolution. SN was the cultural condition that produced the highest level of tryptophan (4.25 mg L^−1^). N-type grapes also showed higher levels than the reference samples in the post-harvest period (3.71 mg L^−1^ vs. 3.54 L^−1^). Therefore, the effect on tryptophan level caused by cultural practices in which nitrogen supplements are applied is still apparent even in the post-harvest period, particularly for SN treatment.

## 3. Materials and Methods

### 3.1. Grape Samples

The vineyard used in this study is located in the area of Jerez (Spain). The maturation periods for Verdejo grapes under five different cultural practices and the reference were evaluated using three replicates per cultural method with the following features:

Reference (R): the reference for comparison with other experimental treatments.

Cover crops (CC): the introduction of cover crops in the fall period, namely *Lolium perenne* and *Hordeum vulgaris*. These crops were removed prior to veraison.

Leaf removal (LR): after flowering (approximately one week later), six leaves were removed mechanically from the clusters.

Sulfur and nitrogen in leaves (SN): a dose of urea and sulfur was applied to the leaves of the vines at two different times. The application of crystalline urea, in doses of 10 Kg N per hectare, and elemental sulfur, at a dose of 5 Kg per hectare, was carried out. The application was divided into two treatments (9 and 5 days before the first sample date). These times correspond to 30% and 100% of the grape veraison.

Specific pruning (P): vines under these conditions retained the same vine structure as the reference, although the amount of grapes was modified because winter pruning was applied. Specifically, those vines that had six to eight additional buds owing to two spurs were substituted by two canes.

Nitrogen on leaves (N): application of a dose of urea on the vine leaves at two different times. Crystalline urea was sprayed at a dose of 10 Kg N per hectare. The application was divided into two treatments (9 and 5 days before the first sample date). These times correspond to 30% and 100% of the grape veraison.

A total of 36 vines for each technique were used to evaluate a representative sample of the grape evolution. A total of 18 grapes from six different clusters were taken for each vine.

Grape juice was obtained using a KitchenAid^®^ system (Countertop Appliances, Benton Harbor, U.K.), which allows a more rapid sample processing than pressing grapes manually.

The technological maturity (sugar/acid) and tryptophan content from the time of 100% veraison to the post-harvest time were analyzed.

A 22-day study was carried out with six sampling points. The research involved five sampling points during the regular ripening period plus one sampling point in the post-harvest period. Information about the sampling points and about the maturity characteristics for each technique and for each day is provided in Table 1. All determinations were carried out in duplicate.

### 3.2. Determination of Tryptophan

Analyses of grape juice were carried out on an ACQUITY UPLC^®^ H-Class system coupled to an ACQUITY UPLC^®^ Photodiode Array (PDA) detector, and the system was controlled by Empower™ 3 Chromatography Data Software (Waters Corporation, Milford, MA, USA). The PDA was set in the wavelength range of 200–400 nm for the 3D scan, with a data collection rate of 40 pts s^−1^ for compound identification. For compound quantification, the PDA detector was set at a fixed wavelength for a 2D scan with a data collection rate at 80 pts s^−1^ at the maximum absorbance of tryptophan and at 278 nm for peak integrations. The identification of tryptophan in the samples was achieved initially by comparing the retention times and UV absorption maxima with those of the standard and then by running spiking procedures with the pure standard.

A volume of 3 μL of sample was injected, and the analysis was carried out at a temperature of 47 °C on a reverse phase Acquity UPLC^®^ BEH column (100 mm length; 2.1 mm I.D.; 1.7 μm particle size) (Waters). The mobile phases were acidified water (2% acetic acid) (solvent A) and 2% acetic acid in acetonitrile (solvent B), and a flow-rate of 0.6 mL·min^−1^ was used.

**Figure 4:**
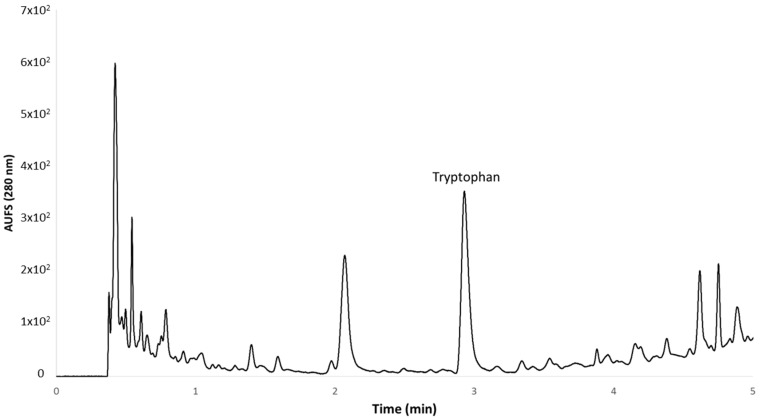
Typical chromatogram from a grape sample during the ripening period.

The gradient used for the separation was as follows: 0 min 0% B, 1.00 min 0% B, 3.00 min 5% B, 4.00 min 10% B, 4.50 min 10% B, 5.00 min 20% B, 7.00 min 20% B, 8.00 min 30% B, 10.00 min 30% B, 11.00 min 100% B. Under these conditions, the retention time for tryptophan was 2.840 min (Figure 4).

HPLC-grade methanol, acetic acid and acetonitrile were purchased from Merck (Darmstadt, Germany). Tryptophan was obtained from Sigma Aldrich (St. Louis, MO, USA). Water was purified with a Milli-Q purification system (Billerica, MA, USA). A stock standard solution of tryptophan was prepared in aqueous methanol 50:50 (*v*/*v*) and stored in a freezer at −32 °C.

The quantification of tryptophan was carried out by integrating the area of the peaks at 278 nm, with standard solutions ranging between 0.2 and 25 mg L^−1^ (12 points) and a coefficient of determination (*R*^2^) of 0.9998. (Limit of Detection (LOD): 0.35 mg L^−1^ and Limit of Quantification (LOQ): 1.18 mg L^−1^). All determinations were carried out in duplicate.

## 4. Conclusions

The cultural techniques that involve the application of nitrogen led to higher levels of tryptophan at the harvest day when compared to other cultural techniques. The application of nitrogen without sulfur produced a faster effect on the level of tryptophan. However, the use of nitrogen plus sulfur gave rise to the highest levels during the post-harvest period. A period of around 20 days was required for the grapes to show clear differences in comparison to grapes from the reference cultural practices.

As far as other cultural practices are concerned, the leaf-removal technique led to a similar maturity to the reference, although the tryptophan content decreased the most in the last stage. In contrast, the cover crop cultural conditions gave rise to higher levels of tryptophan from the beginning to the end of the maturation process when compared to the reference grapes, despite these levels decreasing during the process.

## Figures and Tables

**Table 1:** Levels of sugars (Baumé degrees), total acidity (g/L tartaric acid equivalent), and average grape weight (g/100 grapes) during the ripening period.

Day	Parameters	CC	LR	R	SN	P	N
1	Sugars	10.6	10.5	9.9	9.9	10.3	10.1
	Total acidity	6.64	7.04	5.27	5.97	5.65	6.04
	Average weight (100 grapes)	187.1	184.9	179.1	187.3	186.2	179.1
8	Sugars	11.0	11.0	10.7	10.8	10.8	9.8
	Total acidity	4.81	4.63	4.83	5.03	4.80	4.87
	Average weight (100 grapes)	184.2	178.5	183.9	187.1	190.8	169.6
11	Sugars	11.3	11.1	10.6	10.7	11.0	10.4
	Total acidity	5.13	5.17	4.88	4.85	4.91	4.82
	Average weight (100 grapes)	182.0	186.3	169.1	192.5	183.9	181.9
14	Sugars	11.2	11.0	11.5	11.0	11.3	10.3
	Total acidity	4.37	4.53	4.61	4.55	4.44	4.53
	Average weight (100 grapes)	184.4	180.6	182.8	177.7	190.3	168.1
16 (Harvest)	Sugars	11.9	11.3	11.5	11.4	11.9	10.9
	Total acidity	4.62	4.76	4.62	4.71	4.55	4.78
	Average weight (100 grapes)	182.8	167.5	167.0	173.7	178.0	160.0
22	Sugars			12.1	12.1		12.5
	Total acidity			5.19	4.97		5.03
	Average weight (100 grapes)			177.8	147.3		174.0
